# Should Data from Demographic Surveillance Systems Be Made More Widely Available to Researchers?

**DOI:** 10.1371/journal.pmed.0050057

**Published:** 2008-02-26

**Authors:** Daniel Chandramohan, Kenji Shibuya, Philip Setel, Sandy Cairncross, Alan D Lopez, Christopher J. L Murray, Basia Żaba, Robert W Snow, Fred Binka

## Abstract

Background to the debate: Demographic surveillance—the process of monitoring births, deaths, causes of deaths, and migration in a population over time—is one of the cornerstones of public health research, particularly in investigating and tackling health disparities. An international network of demographic surveillance systems (DSS) now operates, mostly in sub-Saharan Africa and Asia. Thirty-eight DSS sites are coordinated by the International Network for the Continuous Demographic Evaluation of Populations and Their Health (INDEPTH). In this debate, Daniel Chandramohan and colleagues argue that DSS data in the INDEPTH database should be made available to all researchers worldwide, not just to those within the INDEPTH Network. Basia Żaba and colleagues argue that the major obstacles to DSS sites sharing data are technical, managerial, and financial rather than proprietorial concerns about analysis and publication. This debate is further discussed in this month's Editorial.

## Abstract 2

Chandramohan and colleagues argue that DSS data should be made available to all researchers worldwide, while Żaba and colleagues discuss the major obstacles to DSS sites sharing their data more widely.

## Daniel Chandramohan, Kenji Shibuya, Philip Setel, Sandy Cairncross, Alan D. Lopez, and Christopher J. L. Murray's Viewpoint: Demographic Surveillance Data Should Be Considered a Global Public Health Good

Vital statistics on births, deaths, and causes of death are essential for guiding policy, planning, and evaluation of development programmes in all sectors, and particularly in the health sector. Health development is facilitated by critical and incisive analyses of a population's disease burden and determinants of health. But policy and programme planning in many developing countries is severely hampered by the lack of representative vital statistics, data on disease burden, and critical analyses of the limited data that are available. This state of relative ignorance about disease burden and determinants of health is regrettable and unacceptable when there is a network of demographic surveillance systems (DSS) operating throughout sub-Saharan Africa and Asia.

### What Type of Data Do DSS Sites Collect?

A DSS consists of structures and standard operating procedures to monitor vital events (births, deaths, and migration) in a well-defined unit of population. In many DSS sites, data on contextual, epidemiological, socioeconomic, cultural, and health systems determinants of health are also collected periodically. Most DSS sites were initially established as part of large-scale intervention trials. Over the past decade, recognising the growing demand for robust estimates of vital events and determinants of health, the primary objective of DSS evolved to include: (1) the production of timely and reliable population-based health information to support evidence-based health policies, and (2) the monitoring and evaluation of health interventions in settings where the routine health information and vital registration systems are weak.

At present there are 38 DSS sites (26 in Africa, ten in Asia, one in Oceania, and one in Central America) in 19 countries coordinated by the International Network for the continuous Demographic Evaluation of Populations and Their Health (INDEPTH) [1]. There are other DSS or major sample systems that use similar methods to DSS that are not members of INDEPTH (e.g., India's Sample Registration System [2] and China's Disease Surveillance Points system [3]), and a few others have been closed down due to lack of funds (e.g., the Tanzania Adult Morbidity and Mortality Project [4]).

### Suboptimal Access to DSS Data Impedes International Research

DSS sites under the stewardship of INDEPTH have made significant progress in producing and disseminating mortality, fertility, and morbidity data, building research capacity, and refining the methodology of demographic surveillance [5, 6]. Furthermore, DSS sites have been pivotal in generating new knowledge on appropriate interventions to reduce disease burden and to improve health [7]. The data from the DSS sites are disseminated through peer-reviewed publications in journals, occasional monographs, discussion papers, and reports on Web sites [6, 8]. However, one has to question whether the access to these important data sources is optimal to meet the ever increasing need for health information for planning, monitoring, and evaluation and for public health research. We believe lack of access to these data by “external” researchers and public health practitioners is impeding international health development and promotion by precluding critical analyses and different perspectives around key questions for global health development.

Currently, the INDEPTH Network and its member sites (the 14 member sites that have their own Web site) do not state their policy on access to DSS data, and there are no links to DSS databases. Attempts to access DSS data from specific sites in the past have been unsuccessful (A. D. Lopez, personal communication). It appears that access to the INDEPTH databases is restricted to a limited number of investigators and collaborators for an indefinite period. Furthermore, some DSS sites are not enthusiastic about collaborative studies if a sponsor insists on open access to the data after publication of the results (D. Chandramohan, personal communication). This raises the concern that there may be a tendency to “protect” the databases to reduce the risk of “exploitation” by “external” researchers.

The main reasons for this restricted access to the DSS data appear to be: (1) to protect the ownership and intellectual property rights of the investigators; (2) to help offset the costs of maintaining DSS sites; (3) to retain confidentiality of individual participants; and (4) to minimise the risk of misinterpretation of data. Although some of these reasons are of genuine concern, it is legitimate to question whether they are sufficient to restrict access to this invaluable source of data indefinitely, particularly when these obstacles can be overcome by appropriate use of restrictions. Precedents and protocols exist for addressing several of these concerns around data access, and have worked well in the cases where they have been applied. The procedures and mechanisms that are used to provide wider access to data from clinical trials, and to data from demographic and health surveys (DHS), for example, after a certain period of exclusive rights to the investigators, could be adapted by DSS sites. In other words, “external” researchers would be granted a similar level of access to the DSS data as to clinical trial and DHS data, with similar and sufficient conditions to protect intellectual property rights and the confidentiality of participants [9, 10].

### The Benefits of Sharing DSS Data

Wider access to DHS and multiple indicator cluster surveys data has produced important observations on disease burden and public health interventions [11, 12]. It is equally likely that provision of access to DSS data would lead to more innovative analyses and increase their relevance for national and regional health development efforts. For example, the DSS data would provide direct evidence on the relationship between contextual factors and the levels, patterns, and causes of death for several populations in Africa in particular, where epidemiological information is scarce.

Although the DSS data do not come from a random sample of the reference population, detailed analyses of data from well-defined populations could inform our understanding of relationships between key aspects of population health. By illuminating these relationships, DSS data combined with other, more representative sources of data have the potential to contribute to major global health development initiatives, such as monitoring progress in achieving the health-related millennium development goals and assessing equity in health [13]. However, the millennium development indicators across the DSS sites need to be systematically analysed and disseminated periodically. This would help to realise the potential of DSS data for understanding the generalisability of results from DSS to other parts of the developing world.

Fertility and mortality data from 15 DSS sites and model life tables based on these data have been published [1]. Cause-specific mortality rates from 1999–2002 from 12 DSS sites [14], and site-specific trends in mortality in specified periods of time have been reported [15, 16]. However, there is often a four to five year time lag for summary mortality statistics to be available in the public domain. Furthermore, DSS data are not part of the routine health information system in most countries, and there are no defined time points for updating DSS data available in the public domain. In order to optimise their use, DSS data need to be considered as a “global public health good”. Facilitating wider access after a limited ownership period would encourage investigators to take all possible measures to ensure high-quality data and to disseminate findings in a timely manner because other “external” investigators would eventually analyse the data. The continuous appraisal of data and the dialogue between producers of data and “external” investigators would increase the scope of analyses, interpretation, and use of DSS data that in itself is a key factor in refining data collection tools and methods and leads to a virtuous cycle of improved data quality in the future.

### A Balance Between Data “Ownership” and “Exploitation”

Clearly a balance between “ownership” and “exploitation” of data needs to be maintained. One way to optimise the use of these data sources is to define a maximum time limit (18–24 months) for “private” ownership of data. After this ownership period, datasets should be available for further analysis by any bona fide research groups, as with DHS data. The experience with the Human Genome Project, where the recognition of the public good aspect of the sequencing of the human genome led investigators to agree to put their sequences in the public domain after 48 hours, shows that concerns over private ownership can be overcome for the public good. A relatively longer time limit of 18–24 months for providing access to DSS data is a compromise between creating positive incentives for investigators who spend time collecting high-quality data and contributing to the global public good from widespread access.

In 2006, the Wellcome Trust hosted a funders' forum entitled “Harnessing Evidence from DSS Sites for Better Health” (http://www.indepth-network.org/news/bulletin200606.htm). The forum concluded that “data sharing, both among the sites and with external investigators is vital for realising INDEPTH's vision and a carefully titrated policy of data-sharing and co-authorship would progressively lead to shared analysis and give access to external investigators”. Such a policy needs to be accompanied by the necessary resources and efforts to support an increase in the capacity of sites to analyse and disseminate data promptly. Sponsors of DSS and development agencies should ensure that their financial support includes a commitment to develop and maintain a repository of DSS datasets and to enforcing the procedures for accessing the datasets. This support should also mandate defined time points for updating the DSS data for national and sub-national stakeholders. This would be an enormous contribution to building the evidence base for public health that could be made for little additional cost to DSS operations.

Although some of the key users of DSS data are the health planners and policy makers of national governments, most of the DSS sites in Africa receive very limited or no financial support from these governments. National governments should be strongly encouraged and supported to invest in DSS and to incorporate DSS data within their routine health information systems. Demographic surveillance data is a global public health good, and all stakeholders of DSS sites should move towards optimising the use of data for better health outcomes.

## Basia Żaba, Robert W. Snow, and Fred Binka's Viewpoint: There Are Major Technical Obstacles to DSS Data Sharing

Wider public access to primary data has become a clarion call in the bio-medical field, since the highly successful example of the 1996 Bermuda Principles developed by the International Human Genome Sequencing Consortium [17]. These principles called for the automatic, rapid release of sequence assemblies of 1–2 kb or greater to the public domain. Funding agencies such as the Wellcome Trust are extending the requirement for researchers to provide data sharing plans to those working in public health epidemiology, including demographic and behavioural sciences [18]. In their Viewpoint above, Daniel Chandramohan and colleagues fail to point out that many scientists working in DSS actively welcome the new interest in data sharing and the opportunities presented for pursuing new research collaborations to enhance the value of their data. Our Viewpoint addresses the nature of the real obstacles to data sharing by DSS sites.

### Why Are All the DSS Sites in Developing Countries?

Developed countries do not need DSS because they rely on birth and death registration for estimating death rates and the distribution of deaths by cause, and on sophisticated data linkage schemes such as cancer registries, electronic patient record databases, and official resources such as the United Kingdom Longitudinal Study [19] for community-based research. In contrast, the World Health Organization's health indicator database [20] shows that only five African countries have vital registration systems covering more than 25% of the population. Hence in many African countries there is much more reliance on data generated by regular censuses in DSS sites (often involving the collection of biological specimens and detailed behavioural information) and documentation of births, deaths, and in- and out- migrations linked to historical characteristics of individuals, families, and households. Well-run DSS are an excellent source of data for complex health studies of risk factors and interventions that require longitudinal follow-up, but since most DSS are small, unrepresentative “population laboratory” research projects, they do not necessarily provide suitable data for national estimates of burden of disease. Governments in developing countries need to prioritise the development of representative vital registration systems even more than investment in DSS [21].

### Data Flows: Free Trade or Fair Trade?

Developing country scientists want to move away from being primary producers of data for developed country scientists to analyse—they do not wish to remain hewers of data and drawers of protocols. There is an urgent need to enable scientists in the south to play an equal role in the analysis of data they gather, to support their national governments in the science–policy interface, and to develop science careers through appropriate citation in internationally peer-reviewed journals [22]. Young scientists, institutions, and research groups working on assembling demographic and epidemiological data in DSS sites in Africa need to be able to publish their work. We need to enable this to happen before complaining about Northern partners being unable to access data that were considerably harder to collect than identical data from national statistics offices in Canberra and Washington, where 24-hour uninterrupted utilities (such as electricity and Internet access) are taken for granted. Unless there is a major effort to build the capacity of African public health science, the value placed on collecting primary data will diminish as it becomes recognised that there is no career compensation. Who will want to collect data under difficult conditions, manage databases with unreliable power supplies, and train enthusiastic, unqualified high school graduates in the rudiments of computer programming? Free trade in data may signal the birth of the data miner but also the death of the field epidemiologist.

### Recognising the Technical Obstacles

The major obstacles to DSS sites sharing data are technical, managerial, and financial rather than proprietorial concerns about analysis and publication. Many sites are struggling with data management systems unchanged since the projects started many decades ago. These sites do not have the critical mass of programmers and data managers to support the required improvements in data architecture, error reconciliation, and provision of meta-data (information describing the data) for unambiguous interpretation of variables that would be required for efficient data sharing [23]. Because of the piece-meal way that many DSS sites are funded, hosting a variety of different studies that take advantage of their core data collection, many have acquired legacy datasets that are not fully documented and not well integrated in their databases. An enormous programming effort will be needed in many cases to write data dictionaries and edit the data to comply with currently accepted norms. New staff will have to be hired and trained to set up data-sharing systems and manage user requests. Advisory board and steering committee structures must be created to ensure appropriate and legitimate use of data, protecting against commercial interest and exploitation and guaranteeing appropriate citation and acknowledgement to funding sources if data are used [24]. The privacy of the communities under study will need to be carefully protected—for example by removing from public use geographic identifiers that would enable household locations to be identified. Datasets will have to be constructed in such a way that identification of individuals with an uncommon constellation of characteristics is not possible.

Such obstacles need to be addressed by funding agencies, by research partners of developing country DSS sites, and by wealthy developed country research institutions that would like to access these data. We call on these bodies to consider creating special funding and training mechanisms to allow DSS sites to take on and develop the technical staff needed to improve their data management so that effective data sharing becomes feasible.

### The Pioneering Role of Networks

Chandramohan and colleagues singled out the INDEPTH collaborative network formed by the developing country DSS sites for particular criticism as an obstacle to data sharing. This is unfortunate, as INDEPTH does not itself own or generate any data but actually represents the first tentative data sharing steps made by DSS sites, and was formed without prompting from funders or Northern universities. It is not surprising that these first steps led to the “comfort zone” of sharing data with each other: INDEPTH provided a supportive environment in which developing country scientists working in DSS sites could learn the analytical skills and methodological techniques needed for publication, and launched a series of its own peer-reviewed monographs in which the sites are able to compare results on specific topics [25]. The network has been instrumental in showing DSS sites the benefits of mutual collaboration, and is currently developing software and meta-data definition systems that will make it easier for sites to translate their data into mutually compatible forms to facilitate sharing with each other and ultimately with other collaborators.

### We Need Wider Sharing of All Public Health Data

DSS datasets should not be highlighted as a special case for concern. It is just as difficult to access some other datasets that were created specifically for public use. National survey data funded by the United Nations and the bilateral agencies may not be available for public access for several years after their collection, despite being generated with global taxpayers' money. Indeed, data collections compiled by some of the authors in Chandramohan and colleagues' Viewpoint are not publicly available—for example, the primary input data behind the Global Burden of Disease study are not available for public use and scrutiny [26, 27], and the World Health Survey data collected in 2002 were not made available until 2007 [28, 29]. Timely access to public health data on the principle that these are global public goods is *not* simply a DSS/INDEPTH issue, but should extend to censuses, surveys, registration data, and health facility data collected in developed and developing countries. Creating the capacities to assemble quality-controlled data, undertake research enquiries using national and regional data, and build mutual trust between Northern and Southern scientists is critical to the future of health and population sciences in the developing world. Failure to recognise this will signal the decline of scientists prepared to develop careers in this area of public health and the end of any meaningful data collection for anyone to analyse.

## Chandramohan and Colleagues' Response

Basia Żaba and colleagues appear to be in broad agreement with our Viewpoint about the need for easier access to public health data, including DSS data. They raise three main concerns with respect to providing wider and more rapid access to DSS data: (1) building local capacity for analysis and dissemination of data; (2) making the structure and content of data amenable for meta-analysis; and (3) protecting data ownership of DSS investigators. Clearly these concerns have to be addressed to make optimum use of DSS data without demotivating DSS sites.

First, Żaba and colleagues report that the INDEPTH Network's capacity-building activities have led to a “comfort zone” of sharing data among the member sites and to publications. We argue that the efforts to build capacity for data analysis and dissemination should involve *all* institutions with technical expertise in order to maximise the public health impact of these data and foster methodological developments.

Second, the wide variation in data systems and structures has been a major obstacle to the sharing and pooling of data, even among DSS sites. The future application of new international standards for verbal autopsy tools [30], as well as for demographic data collection and management, should help to ameliorate this constraint. With adequate financial and technical support, these tools can be adapted and implemented widely.

Third, we agree that ensuring “fairness” to data producers is indeed a necessary part of any commitment to wider access. However, restricting access to basic demographic data on births, deaths, and causes of death is not an appropriate incentive or means to build local capacity. Indeed it is unlikely that the DSS investigators will be demotivated by providing the opportunity for wider collaboration and better use of data. We believe that wider access to DSS data is feasible if all stakeholders are committed to “fair data sharing for maximising public health utility of data”.

The theme of the recent malaria forum sponsored by the Bill & Melinda Gates Foundation was “collaboration, innovation and impact”. We call for a high-level demographic surveillance data forum on the same theme to agree upon a way forward and to harness financial and technical support to build capacity for innovation, better sharing, and utilisation of public health data.

## Żaba and Colleagues' Response

We are pleased to note a subtle change in the stance of Chandramohan and colleagues in their response above. The discussion has now shifted to the need for easier access to *all* public health data while building research capacity and recognition of DSS sites and their scientists.

Technical expertise in data analysis is eagerly sought by DSS sites, especially if it comes from a rooted understanding of the complexities and inter-site variability of data structures, as opposed to pre-conceived “one-size fits all” solutions. The success of networks, such as INDEPTH, in developing methodologies that are widely adopted by member sites depends on promoting interaction between analysts and collectors of data. Colleagues from various universities in the North are already engaged in working with INDEPTH on the development of prototype structures and data description tools to enable data sharing.

One way to ensure “fairness” to data producers and encourage data sharing would be to create an open-access journal specialising in publication of descriptive results from observational studies (such as analyses of mortality risks and cause of death structures) and detailed methodological accounts of collection and management of longitudinal data. Just as PLoS ONE (http://www.plosone.org/) encourages authors to make available code used in modelling and statistical analyses, this new outlet could encourage authors to make available subsets of data used in their published analyses, along with suitable data dictionaries. This would encourage publication and peer review of the kind of basic findings that usually appear only in monographs and reports, and would ensure that all future users of the data would acknowledge the producers by citing the original publication. Sites could thereby share as much or as little data as they wished, and could present them in the form used in the accompanying published analysis, without worrying about conforming to pre-specified structures—although such publication would also gradually encourage structural standardisation.

Finally, we agree that there is a need for a high-level forum on demographic surveillance data, linking data producers, analysts, and users, to discuss collaboration, innovation, and impact. Work initiated by the Health Metrics Network [31] suggests that it would be a good forum to bring together Northern and Southern partner scientists to set an equitable agenda for population-based health sciences.

## Abbreviations

DHS: demographic and health survey
DSS: demographic surveillance system(s)
INDEPTH: International Network for the continuous Demographic Evaluation of Populations and Their Health

## References

[pmed-0050057-b001] INDEPTH Network (2008). INDEPTH demographic surveillance sites. http://www.indepth-network.org/dss_site_profiles/dss_sites.htm.

[pmed-0050057-b002] Census of India (2007). Sample Registration System. http://www.censusindia.gov.in/Vital_Statistics/SRS/Sample_Registration_System.aspx.

[pmed-0050057-b003] Yang G, Hu J, Rao KQ, Ma J, Rao C (2005). Mortality registration and surveillance in China: History, current situation and challenges. Popul Health Metr.

[pmed-0050057-b004] Tanzania Ministry of Health (2004). Tanzania Adult Morbidity and Mortality Project. http://research.ncl.ac.uk/ammp/.

[pmed-0050057-b005] INDEPTH Network (2006). INDEPTH resource kit for demographic surveillance systems. http://www.indepth-network.org/Resource%20Kit/INDEPTH%20DSS%20Resource%20Kit/contents.htm.

[pmed-0050057-b006] INDEPTH Network (2004). INDEPTH model life tables for sub-Saharan Africa. http://www.indepth-network.org/publications/zindpubs/mortality_monograph/INDEPTH_ModelLifeTables.pdf.

[pmed-0050057-b007] Binka F, Hodgson A, Adjuik M, Smith T (2002). Mortality in a seven-and-a-half year follow-up of a trial of insecticide-treated mosquito nets in Ghana. Trans R Soc Trop Med Hyg.

[pmed-0050057-b008] Tanzania Adult Morbidity and Mortality Project (2002). Reports, correspondence, working papers, and conference presentations. http://research.ncl.ac.uk/ammp/project_outputs/reports.html.

[pmed-0050057-b009] MEASURE Demographic and Health Surveys (2008). Download datasets. http://www.measuredhs.com/login.cfm.

[pmed-0050057-b010] UNICEF (2008). MICS2-Datasets. http://www.childinfo.org/MICS2/Mailform.php.

[pmed-0050057-b011] Webster J, Lines J, Bruce J, Armstrong Schellenberg JR (2005). Which delivery systems reach the poor? A review of equity of coverage of ever-treated nets, never-treated nets, and immunisation to reduce child mortality in Africa. Lancet Infect Dis.

[pmed-0050057-b012] Victora C, Fenn B, Bryce J, Kirkwood B (2005). Coverage of preventive interventions and implications for child-survival strategies: Evidence from national surveys. Lancet.

[pmed-0050057-b013] Ngom P, Binka F, Phillips J, Pence B, Macleod B (2001). Demographic surveillance and health equity in sub-Saharan Africa. Health Policy Plan.

[pmed-0050057-b014] Adjuik M, Smith T, Clark S, Todd J, Garrib A (2006). Cause-specific mortality rates in sub-Saharan Africa and Bangladesh. Bull World Health Organ.

[pmed-0050057-b015] Nhacolo A, Nhalungo D, Sacoor C, Aponte J, Thompson R (2006). Levels and trends of demographic indices in southern rural Mozambique: Evidence from demographic surveillance in Manhica district. BMC Public Health.

[pmed-0050057-b016] Baiden F, Hodgson A, Adjuik M, Adongo P, Ayaga B (2006). Trends and causes of neonatal mortality in the Kassena-Nankana district of northern Ghana, 1995–2002. Trop Med Int Health.

[pmed-0050057-b017] Waterston R, Sulston JE (1998). The human genome project: Reaching the finish line. Science.

[pmed-0050057-b018] Wellcome Trust (2007). Wellcome Trust policy on data management and sharing. http://www.wellcome.ac.uk/doc_WTX035043.html.

[pmed-0050057-b019] Centre for Longitudinal Study Information and User Support (2007). What is the LS. http://www.celsius.lshtm.ac.uk/.

[pmed-0050057-b020] World Health Organization (2008). WHO statistical information system database. http://www.who.int/whosis/en/.

[pmed-0050057-b021] Mathers CD, Ma Fat D, Inoue M, Rao C, Lopez AD (2005). Counting the dead and what they died of: An assessment of the global status of cause of death data. Bull World Health Organ.

[pmed-0050057-b022] Baiden F, Hodgson A, Binka FN (2006). Demographic surveillance sites and emerging challenges in international health. Bull World Health Organ.

[pmed-0050057-b023] Data Documentation Initiative (2007). About the specification. http://www.ddialliance.org/codebook/index.html.

[pmed-0050057-b024] Lowrance WW (2006). Access to collections of data and materials for health research: A report to the MRC and the Wellcome Trust. http://www.wellcome.ac.uk/doc_WTX030843.html.

[pmed-0050057-b025] INDEPTH Network (2005). Publications from INDEPTH. http://www.indepth-network.org/publications/indepth_publications.htm.

[pmed-0050057-b026] Ezzati M, Lopez AD, Rodgers A, Murray CJL (2004). Comparative quantification of health risks: Global and regional burden of diseases attributable to selected major risk factors.

[pmed-0050057-b027] World Health Organization (2002). The world health report 2002: Reducing risks, promoting healthy life. http://www.who.int/whr/2002.

[pmed-0050057-b028] Üstün TB, Chatterji S, Villanueva M, Bendib L, Çelik C (2001). WHO multi-country survey study on health and responsiveness 2000–2001. World Health Organization. http://www.who.int/healthinfo/survey/whspaper37.pdf.

[pmed-0050057-b029] World Health Organizatoni (2007). World health survey. http://www.who.int/healthinfo/survey/en/.

[pmed-0050057-b030] World Health Organization (2007). Verbal autopsy standards: Ascertaining and attributing cause of death.

[pmed-0050057-b031] Health Metrics Network (2007). Stepping stones to improving the monitoring of vital events: A resource kit for strengthening national vital statistics systems. http://www.who.int/healthmetrics/tools/logbook/en/move/web/index.html.

